# Targeting oxidative stress-MMP-2 signaling in hypertension-induced cardiovascular remodeling and dysfunction

**DOI:** 10.3389/fphar.2026.1873305

**Published:** 2026-07-29

**Authors:** Marcela M. Blascke de Mello, Laena Pernomian, Luan V. R. Ramos, Michele M. Castro

**Affiliations:** 1 Department of Pharmacology, Ribeirao Preto Medical School, University of Sao Paulo, Ribeirao Preto, Brazil; 2 Department of Cell Biology and Anatomy, School of Medicine Columbia, University of South Carolina, Columbia, SC, United States

**Keywords:** antioxidants, cardiovascular dysfunction, hypertension, MMPs, oxidative stress

## Abstract

Excessive reactive oxygen and nitrogen species contribute to maladaptive structural and functional changes in the cardiovascular system in hypertension, including extracellular matrix proteolysis, vascular smooth muscle cell proliferation and arterial stiffness. A strong link exists between oxidative stress and increased matrix metalloproteinases (MMP) activity in inflammatory cardiovascular diseases. In particular, MMP-2 plays a central role in proteolytic processes in the vasculature and heart, contributing to maladaptive remodeling and dysfunction in hypertension. Oxidative stress regulates MMP expression and activity through transcriptional, post-transcriptional and post-translational mechanisms, including pathways mediated by peroxynitrite (ONOO^−^)-dependent S-glutathiolation. This review provides a comprehensive overview of experimental and clinical evidence supporting the effects of enzymatic and non-enzymatic antioxidants, including dietary compounds such as polyphenols, carotenoids and vitamins, as well as pharmacological agents such as doxycycline, NADPH oxidase inhibitors, nitrite and hydrogen sulfide donors. Overall, antioxidants attenuate oxidative stress, improve nitric oxide bioavailability, reduce MMP activity and may ameliorate cardiovascular remodeling and dysfunction in experimental hypertension, although more clinical trials need to be performed to better translate such benefit effects in humans. A better understanding of antioxidant-mediated modulation of MMPs may support the development of novel therapeutic strategies to prevent or reduce hypertensive cardiovascular diseases.

## 1 Oxidative stress in hypertension-induced vascular remodeling and dysfunction

Arterial hypertension is a noteworthy health problem and one of the leading causes of death worldwide (Palaniappan et al., 2026). It is a multifactorial disease that involves genetic and epigenetic factors associated with lifestyle, such as diet, sedentarism, smoking, alcohol and weight gain (Virani et al., 2021). Hypertension leads to sustained increase in the blood pressure and is a risk factor for the development of several cardiovascular diseases, such as heart and renal failure, myocardial infarction, stroke and chronic vascular remodeling (Raftopoulos et al., 2015; V Chobanian et al., 2003). The maladaptive arterial remodeling occurs as either a cause or a consequence of hypertension and is characterized by extracellular matrix (ECM) remodeling and increased vascular smooth muscle cells (VSMC) proliferation or migration (Belo et al., 2015). There are two main types of arterial remodeling in hypertension: eutrophic and hypertrophic. Reduced lumen diameter with normal media thickness and increased media per lumen ratio (M/L) are characteristics of eutrophic remodeling, which generally occurs in the resistance arteries. As VSMC switch from contractile to migratory phenotype, they overlap in the vascular luminal space and reduce its size (Harvey et al., 2015; Lemarié et al., 2010). It is important to note that VSMC has different migratory phenotype as mesenchymal-, fibroblast-, macrophage, osteogenic- and adipocyte-like, in which they switch after triggered by specific stimuli (Yurdagul, 2022). Then, increased vasoconstriction contributes to enmeshing VSMC in the ECM and impairs arterial vasodilation. Increased M/L is an important marker to characterize arterial remodeling in humans and precede organ damage in essential hypertension (Park and Schiffrin, 2001; Mulvany, 2008; Castorena-Gonzalez et al., 2014). On the other hand, a considerable increase in the arterial wall thickness, cross sectional area (CSA) and M/L are seen in the hypertrophic remodeling, which mostly affects the conduit arteries such as the aorta. This remodeling is associated with ECM proteolysis and concurrently increased deposition of collagen in the medial layer, which may lead to stiffness (Redón et al., 2003).

The maladaptive vascular remodeling and dysfunction in hypertension are generally associated with an excessive level of reactive oxygen and nitrogen species (RONs) that trigger several proliferative signaling pathways in VSMC (Montezano et al., 2014). RONs are products of the metabolism of many pro-oxidant enzymes, such as the diverse isoforms of NADPH oxidase (or NOX), xanthine oxidase and the uncoupled endothelial nitric oxide synthase (eNOS). In fact, oxidative stress oxidizes the tetrahydrobiopterin (BH_4_), an eNOS cofactor, and contributes to shift the dimeric eNOS to its monomeric isoform that synthesizes superoxide anion (O_2_^−^) instead of nitric oxide (NO) (Pinheiro and Oliveira-Paula, 2020). Hydroxyl radical (•OH), nitrogen dioxide (NO_2_), hydrogen peroxide (H_2_O_2_) and lipid-derived radicals, such as alkoxyl (RO•), peroxyl (ROO•) are also characterized as RONs. The mitochondria are another important source of RONs in hypertension and other cardiovascular diseases because some of its constituents, for example, the electron transport chain proteins, the inner membrane phospholipids, especially cardiolipin, and DNA (mtDNA) are damaged by oxidative stress (Ma et al., 2024). Then, a vicious cycle is created whereby oxidative stress causes damage to the mitochondria, and mitochondrial dysfunction is generally associated with impaired oxidative phosphorylation and electron leakage from complexes I and III (Zong et al., 2024; Camargo et al., 2025). This leakage of electrons reacts with oxygen, thus generating even more RONs, as the O_2_^−^.

The formation of RONs is contra balanced by antioxidants. Vascular superoxide dismutase (SOD) converts O_2_^−^ in H_2_O_2_, which in turns, is transformed in H_2_O and O_2_ by catalase (CAT). Either an increase in the pro-oxidant enzymes activity or a reduction in the antioxidants leads to oxidative stress in hypertension, which occurs in all organs, including the vasculature. Excessive RONs contribute to stimulate VSMC migration and/or apoptosis in addition to trigger vascular inflammation and ECM remodeling in many models of hypertension (Harvey et al., 2015; Schulz, 2007). Peroxynitrite (ONOO^−^) is one of the main harmful RONs and is rapidly formed by reacting O_2_^−^ with NO. Increased ONOO^−^ in the vasculature contributes to hypertension-induced increased contraction and reduced endothelium-dependent relaxation, in part, by decreasing NO (Pinheiro and Oliveira-Paula, 2020; Maron and Michel, 2012).

Moreover, ONOO^−^ contributes to activate the Ras homolog family member, A/Rho-associated coiled-coil containing protein kinase (RhoA/ROCK), during hyperglycemia in rat coronary arteries, thus decreasing vasomotor responses (Aleksandrowicz, 2025). ONOO^−^ also directly reacts with the electron-rich groups in many proteins and lipids, such as sulfhydryl sites (Touyz and Schiffrin, 2004), iron-sulfur centers and zinc-thiolates thus contributing to post-translational modifications (Levy, 2005; Ferroni et al., 2006; Kobori et al., 2007), that alter function of many proteins and influence their biological responses in cardiovascular diseases. In this context, ONOO^−^ contributes to activate a zinc-dependable group of proteases known as matrix metalloproteinase (MMP)s, especially MMP-2, by reacting with the sulfhydryl ligation between its cysteine in the propeptide domain with its catalytic site. This posttranslational modification is called S-glutathiolation and may be crucial for MMP-2 intracellular effects in many cardiovascular diseases, including hypertension (Ferroni et al., 2006; AC, 2006). Grote et al. showed that after mechanically stretching VSMC from p47phox NADPH oxidase knockout mice, there was a significant decrease in the mRNA and protein levels of MMP-2 (Grote et al., 2003). In the next session, we will briefly discuss the importance of MMP-2 activity for developing maladaptive vascular remodeling and dysfunction in hypertension, and how the antioxidants reduce its activity and improve some cardiovascular alterations.

## 2 Oxidative stress as an important regulator of the expression and activity of MMPs in hypertension

MMPs are a class of zinc-dependent endopeptidases that are capable to proteolyze and stimulate the rebuilding of many ECM proteins, like collagen, elastin, fibronectin and laminin in many tissues, including vessels. There are at least 23 MMPs identified in humans (Madz et al., 2019) based on their structure and substrate specificity. MMPs are classified into multiple subfamilies. These include membrane MMPs (also called MT-MMP, like MMP-14, -17, -24 and 25), stromelysins (MMP-3, -10 and -11), collagenases (MMP-1, -8, -13), gelatinases (MMP-2 and MMP-9), matrilysins (MMP-7 and MMP-26) and others MMPs (MMP-12, -19, -20, -21, -22, -23, -27 and 28). The MMP-2 and MMP-9 have a typical structure that comprises three main domains: the propeptide, the catalytic site with zinc ion, and a hemopexin domain, linked to a fibronectin type II-rich sequence, that confers specificity and high affinity for proteolyzing gelatin and type IV collagen in the basement membrane. The 72 kDa MMP-2 is largely found in the VSMC and cardiomyocytes and is activated extracellularly by a complex formed by MMP-14 and the tissue inhibitor of MMP (TIMP)-2. On the other hand, the 92 kDa MMP-9 is an inducible protease that is usually found in inflammatory cells that infiltrate in the vasculature and heart in inflammatory diseases (Visse et al., 2003; Ågren and Auf Dem Keller, 2020).

**FIGURE 1:**
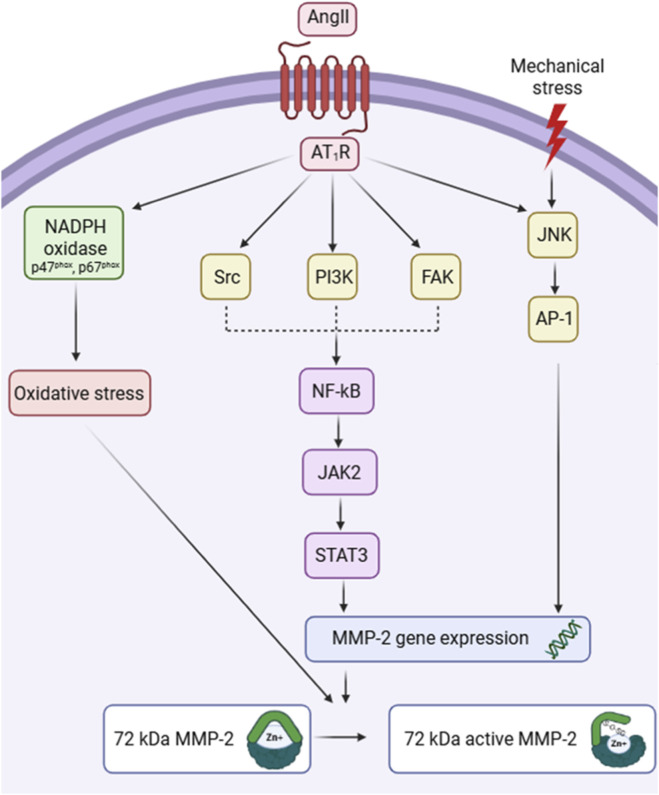
Key signaling pathways linking angiotensin II-induced oxidative stress to MMP-2 expression and activation in the cardiovascular system. Angiotensin II (Ang II) binds to the AT_1_ receptor (AT1R), stimulating multiple intracellular signaling pathways, including NADPH oxidase-derived reactive oxygen and nitrogen species (RONs), Src-family tyrosine kinase (Src), phosphatidylinositol-3-kinase (PI3K), focal adhesion kinase (FAK), and c-Jun N-terminal kinase/transcription factor activator protein-1 (JNK/AP-1). These pathways can converge on redox-sensitive transcription factors, such as nuclear factor kappa B (NFκB), AP-1, and Janus kinase/signal transducers and activators of transcription (JAK2/STAT3), leading to increased MMP-2 gene expression. In parallel, oxidative stress contributes to the post-translational activation of 72 kDa MMP-2, generating the enzymatically active form of 72 kDa MMP-2. Together, these mechanisms increase MMP-2 activity, a key mediator of extracellular matrix remodeling, intracellular proteolysis and cardiovascular alterations associated with hypertension.

Increased levels of RONs upregulate the 72 kDa MMP-2 at transcriptional and post-transcriptional levels, thus increasing gene and protein expression and activity in hypertension (Grote et al., 2003; Viappiani et al., 2009). One of the mechanisms occurs via the nuclear factor kappa-light-chain-enhancer of activated B cells (NFκB), which is activated by oxidative stress in VSMC in hypertension. When NFκB is phosphorylated, it migrates to the nucleus of VSMC and binds to the promoter region of MMP-2 gene, thus increasing its transcription and protein expression. Angiotensin II (AngII), via angiotensin type 1 receptor (AT_1_R), is one of the main mediators of the activation of pro-oxidant enzymes, as NADPH oxidase, in the arteries in hypertension (Figure 1) (Humphrey, 2021). This contributes to form RONs and incites the phosphorylation of NFκB, which increases MMP-2 gene and protein expression, and triggers VSMC migration and hypertrophic remodeling (Humphrey, 2021). AngII also increases the expression of MMP-2 in the vasculature by activating p38 mitogen-activated protein kinase (MAPK), extracellular signal-regulated kinases (ERK) 1 and 2, c-Jun N-terminal Kinase (JNK) and the transcription factor activator protein (AP)-1 (Kopaliani et al., 2014). AngII also stimulates MMP proteolyzes the membrane-bound epidermal growth factor (EGF)-like ligands, which activates EGF receptor (EGFR). Activated EGFR leads to phosphorylation and activation of Janus kinase (JAK)-2 and the signal transducer and activator of transcription (STAT)-3, that regulates inflammatory genes, oxidative stress and ECM remodelling in the vasculature in hypertension (Jiménez et al., 2009). We then summarized these keys signaling pathways that contribute to link oxidative stress and MMP-2 in the cardiovascular system in the Figure 1.

As a counterbalanced pathway, the nuclear factor erythroid 2-related factor 2 (Nrf2) is often activated by transient oxidative or electrophilic stress and moves into the nucleus of VSMC, for example, and increases the expression of SOD, CAT, glutathione peroxidase (GPx), NAD(P)H quinone oxidoreductase 1 (NQO1) and heme oxygenase (HO)-1, thereby decreasing the availability of RONs (Ch et al., 2018). Given that RONs directly regulate MMP-2, the activation of Nrf2 indirectly suppresses MMP-2 by reducing NFκB and AP-1, as well as its post-translational activation via reducing oxidative stress-induced S-glutathiolation (Hiebert, 2021; Shi et al., 2017).

**FIGURE 2:**
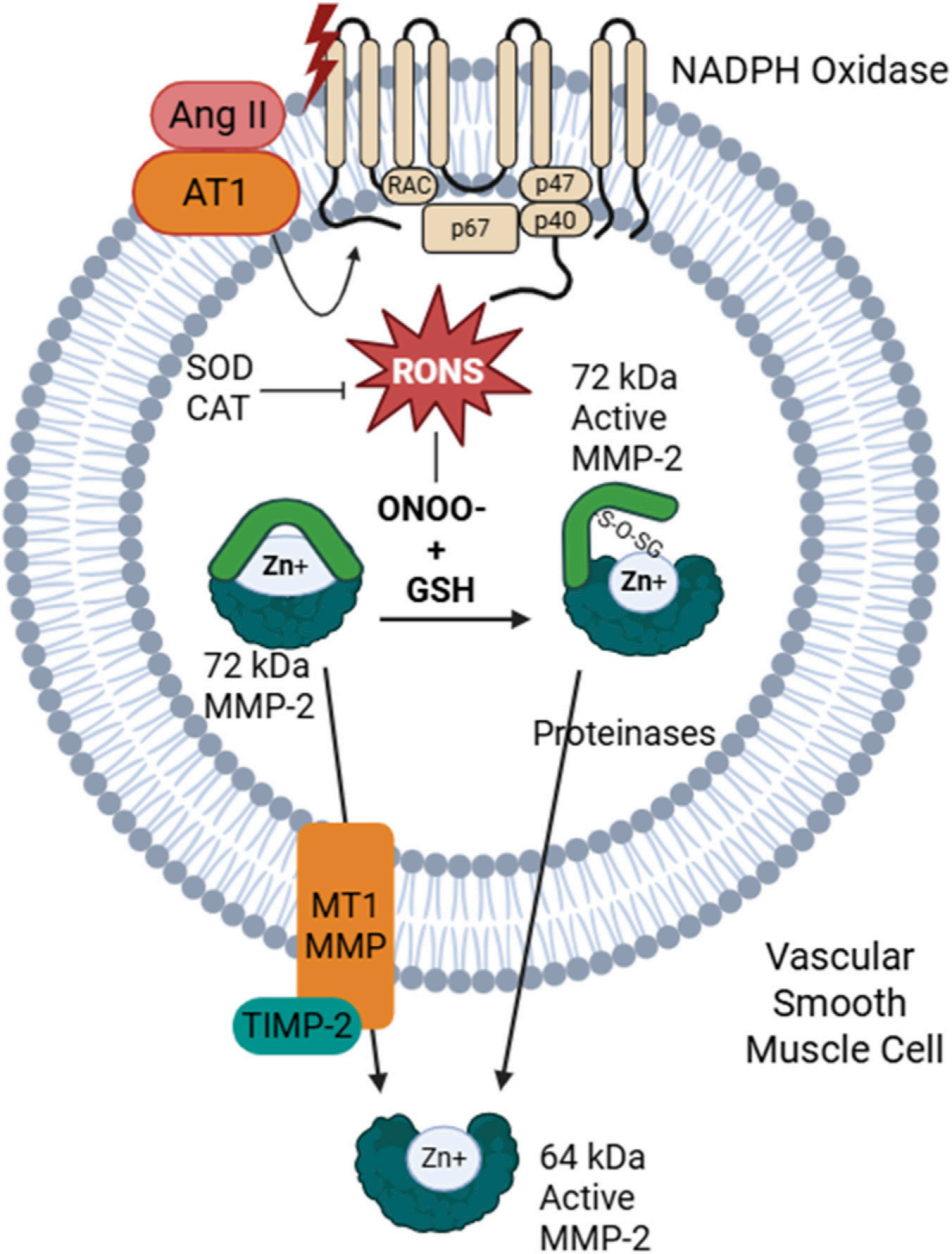
Extra- and intracellular activation of MMP-2. NADPH oxidase (or NOX, which includes p47, p67, p40 and Rac subunits) is activated in the membrane of vascular smooth muscle cells (VSMC) by angiotensin II (AngII), and produces reactive oxygen and nitrogen species (RONs). Antioxidant system like superoxide dismutase (SOD) and catalase (CAT) contra balanced the production of RONs. RONs, especially peroxynitrite (ONOO^−^), contribute to activate the 72 kDa MMP-2 by post-translational changes, including ONOO^−^/glutathione (GSH)-mediated S-glutathiolation, which may result in the proteolysis of intracellular proteins. Additionally, when secreted from the cells, the MT1-MMP/TIMP-2 complex activates 72 kDa MMP-2 by removing the propeptide domain and producing the active 64 kDa. Finally, MMP-2 causes vascular remodeling by functioning extra- and intracellularly, thus proteolyzing many different proteins.

The 72 kDa MMP-2 activity also can be controlled by both proteolytic and non-proteolytic pathways. The usual proteolytic pathway occurs when MMP-2 is secreted from the cells and consists in the cleavage of its propeptide domain from the catalytic site by other proteases, such as MMP-14 and serine proteases, thus reducing the 72 kDa–64 kDa MMP-2. A trimolecular complex of MMP-2, MMP-14 and TIMP-2 is formed, and MMP-14/TIMP-2 acts as MMP-2 ligand and another MMP-14 comes to activate this complex (Figure 2) (Wang et al., 2000). This effect disrupts the binding between the propeptide domain of MMP-2 with the zinc ion in its catalytic site, thus allowing its proteolytic effect on the ECM (Wang et al., 2000). On the other hand, TIMP-2 also has an inhibitory effect on MMP-2 activity, thus finely regulating its proteolytic effect on ECM. A noncovalent bond occurs between the N-terminal portion of TIMP-2 with the catalytic site of MMP-2, which then blocks its access to the substrates. Each TIMPs has preferences to inhibit one specific MMPs; for example, while TIMP-2 prefers inhibiting MMP-2, TIMP-1 inhibits MMP-9 (Visse et al., 2003; Stetler-Stevenson, 2008). TIMP-4 seems to inhibit MMP-2 in maladaptive cardiovascular remodeling and seems to have an intracellular inhibitory effect, especially in the cardiomyocytes in ischemia and reperfusion (I/R) injury (Webb et al., 2006). TIMP-3 is an inhibitor of the ADAMs (Disintegrin-MMP) family, specifically ADAM-17, which is known for cleaving some membrane proteins, such as soluble tumor necrose factor (TNF)-α (Black et al., 1997; Blobel, 2005). ADAMs are a class of transmembrane MMPs that share the zinc-dependent catalytic domain and have similar mechanisms of regulation with MMPs. They have important roles in either controlling the activation of growth factors and cytokines or cleaving and releasing the ectodomain of membrane proteins (Black et al., 1997; Arora et al., 2025).

At posttranslational levels, increased RONs, especially ONOO^−^, increase the activity of MMP-2 in the vasculature in hypertension by causing the S-glutathiolation of its active site. In fact, ONOO^−^ and S-glutathione (GSH) react with the sulfhydryl ligation between the cysteine in the propeptide domain of MMP-2 with the zinc ion in its catalytic site, thus forming a protein complex called disulfide S-oxide (Okamoto et al., 2001). This reaction induces conformational change in the structure of MMP-2, thus making its catalytic site free to cleave proteins even in the presence of the pro-peptide (Figure 2). This activation may occur intracellularly and contributes to the maladaptive remodeling of many cardiovascular diseases (Park and Schiffrin, 2002; Schiffrin, 2004). Other posttranslational modifications that contribute to activate MMP-2 are the phosphorylation and O-GlcNAcylation. The phosphorylation of MMP-2 at serine and threonine residues in the propeptide domain changes its conformation and interferes with its interaction with substrates, as well as its stability and subcellular localisation (Jacob-Ferreira et al., 2013). In myocardial I/R in rats, the phosphorylation of MMP-2 by protein kinase C was associated with reduction of its activity (Sariahmetoglu et al., 2012). On the other hand, alkaline phosphatase contributes to MMP-2 dephosphorylation and increases its activity (Sariahmetoglu et al., 2007). Moreover, phosphorylation of MMP-2 also affects the occurrence of S-glutathiolation and O-GlcNAcylation, as its dephosphorylation facilitates S-glutathiolation *in vitro* (Jacob-Ferreira et al., 2013). O-GlcNAcylation consists in the enzymatic attachment of N-acetylglucosamine (GlcNAc) to serine and threonine residues of MMPs, in the presence of high levels of glucose. However, whether the O-GlcNAcylation increases or reduces the activity of MMP-2 in hypertension is not known. The cross-modulation between oxidative stress and metabolism may represent an important mechanism in the non-proteolytic activation of MMP-2 in cardiovascular diseases (Dozio et al., 2021).

## 3 Contribution of MMPs in hypertension-induced vascular remodeling and dysfunction

The activation of the 72 kDa MMP-2 in vasculature causes hypertrophic maladaptive remodeling and dysfunction in many experimental models of hypertension, such as two-kidney, one-clip (2K-1C) rats (Castro et al., 2008), deoxycorticosterone acetate (DOCA)-salt rats (Watts et al., 2007), and AngII infusion in rodent by osmotic mini-pumps (Barhoumi et al., 2017; Ramos et al., 2025). Increased MMP-2 activity may proteolyze type IV collagen at basement membrane of VSMC, thus contributing to induce migration and hypertrophic remodeling through ECM-integrin signaling activation (Belo et al., 2015). Elastin is another target of MMPs in the arteries during hypertension and, its cleavage, forms the elastin-derived peptides that may cause VSMC proliferation and arterial rigidity (Maurice et al., 2013). Moreover, when elastic fibers are disrupted, the elastic capacity of the arterial wall is weakened, thus being replaced by collagen, leading to stiffness (Maurice et al., 2013; Coccio et al., 2018). Treating 2K-1C rats with doxycycline, a broad MMP inhibitor, attenuated MMP-2 activity in the aorta and hypertrophic remodeling, followed by reduction in VSMC proliferation and the resynthesis of collagen and elastin (Castro et al., 2008). Moreover, doxycycline reversed MMP-2-induced proteolysis of type I collagen in the aortas of 2K-1C rats, thus decreasing the activation of focal adhesion kinase (FAK) and VSMC migration and proliferation, which attenuated hypertrophic remodeling (de Oliveira Neves et al., 2023).

**FIGURE 3:**
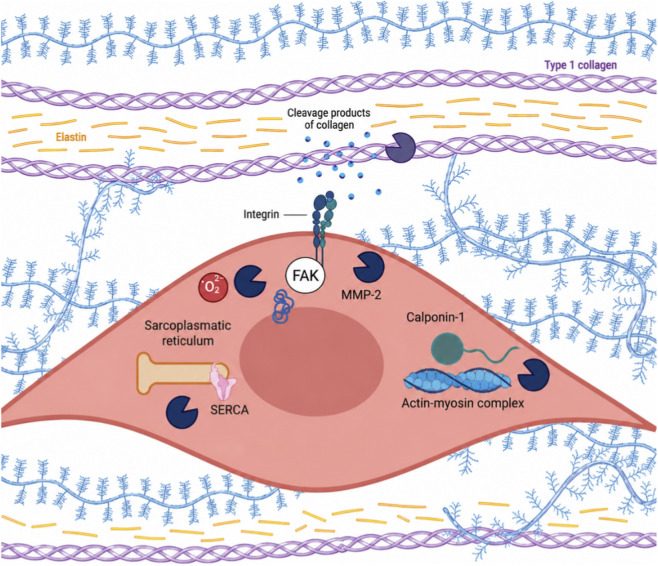
Proteolytic effects of MMP-2 in vascular smooth muscle cells (VSMC) that contribute to maladaptive vascular remodeling in hypertension. Peroxynitrite (ONOO^−^) activates intracellular MMP-2, thus enabling its interaction or proteolytic effect of multiple cytosolic and structural proteins in the VSMC, such as calponin-1 in the contractile machinery (in green), sarco-/endoplasmic reticulum Ca^2+^-ATPase (SERCA, in light pink) in the endoplasmic reticulum, and focal adhesion kinase (FAK) after cleaving type I collagen in the extracellular matrix. These effects may contribute to increase cytoskeletal disorganization, calcium handling and VSMC differentiation/migration. In parallel, the activation of MMP-2 extracellularly contributes to proteolyze type I collagen (in purple) and elastin (in yellow), thus helping the occurrence of maladaptive vascular remodeling. Inhibiting MMP-2 activity by using MMP inhibitors or antioxidants may reduce these proteolytic effects and ameliorates vascular remodeling and dysfunction in hypertension.

Yet, the non-proteolytic 72 kDa MMP-2 activation also contributes to maladaptive vascular remodeling of hypertension (Figure 3). In fact, MMP-2 proteolyzes calponin-1 in the aorta of 2K-1C rats, which leads to VSMC proliferation and increased CSA and M/L (Belo et al., 2016). Treatment of 2K-1C rats with tempol reduced S-glutathiolation of MMP-2 in the aortas and inhibited its proteolytic effect on calponin-1 (Blascke de Mello et al., 2019)**.** Calponin-1 is also proteolyzed by MMP-2 in aortas of endotoxemic (by lipopolysaccharide, LPS) rats, where resulted in hyporeactivity to vasoconstrictors (Castro et al., 2012a). Additionally, by treating 2K-1C rats with doxycycline, MMP-2 activity was inhibited to proteolyze the sarcoplasmic reticulum calcium ATPase (SERCA) in the aortas, thus maintaining the physiological levels of calcium in the cytoplasm and reducing VSMC proliferation and endothelial dysfunction (Blascke de Mello et al., 2024). Part of these MMP-2 effects is shown in the Figure 3. In myocardial I/R injury in rodents, MMP-2 also proteolyzes troponin I, titin and SERCA in cardiomyocytes, thus contributing to cause acute myocardial dysfunction. These deleterious effects of MMP-2 were significantly inhibited by doxycycline or when MMP-2 is knockdown (Cheung et al., 2000; Kandasamy et al., 2010; Roczkowsky et al., 2019). Moreover, increased MMP-2 activity activates the latent form of transforming growth factor (TGF)-β in hypertension by proteolyzing its anchoring proteins such as the latent associated peptide (LAP) and the latent TGF-β binding protein (Annes et al., 2003). Then, activated TGF-β binds to its receptors in the VSMC and stimulates the transcription of collagen and fibronectin, as well as genes for hypertrophy and proliferation (Ruiz-Ortega et al., 2007) Cadherins are also potential targets of MMPs in the VSMC as the use of MMP inhibitors prevented their proteolysis and reduced VSMC migration and proliferation *in vitro* (Uglow et al., 2003).

Doxycycline has inhibitory effects on MMPs activity and is regularly used in diseases associated with oxidative stress and inflammation, such as periodontitis, hypertension and aneurysm (Ceron et al., 2010). Doxycycline can directly bind to the zinc ion in the catalytic domain of MMPs, thus preventing their interaction with the substrates, in addition to downregulate their mRNA levels (Hadjimichael et al., 2020). A randomized, double-blind placebo-controlled study showed that patients who received doxycycline (100 mg, 2x daily) for 2–8 weeks before undergoing carotid endarterectomy showed reduced levels of MMP-1 and -9 in the carotid plaques (Axisa et al., 2002). Another randomized, double-blind placebo-controlled study showed that doxycycline (20 mg, bid) reduced cardiac MMP-2 in coronary artery bypass patients undergoing cardiopulmonary bypass without ameliorating myocardial dysfunction during reperfusion (Schulze et al., 2013). The use of doxycycline as an MMP inhibitor, at 20 mg twice per day, was approved by the US Food and Drug Administration for treating human periodontitis (Engebretson and Hey-Hadavi, 2011). On the other hand, some *in vitro* and *in vivo* studies have been used doxycycline as an antioxidant as scavengers RONs (Dikalov et al., 2014). We will discuss about the antioxidant mechanism of doxycycline in the next session.

Others MMP inhibitors are also used to ameliorate vascular dysfunction in many animal models of hypertension or other inflammatory, cardiovascular diseases. In a rat model of chronic hypoxia, Batimastat reduced MMP-2 activity, thus preventing pulmonary vascular remodeling (Herget et al., 2003). In Dahl salt-sensitive rats, treatment with GM6001 attenuated MMP-2 activity and vascular maladaptive remodeling (Pushpakumar et al., 2013). Moreover, ONO-4817 inhibited MMP-2 *in vitro* (Tanase et al., 2025) and in experimental models of cardiotoxicity (Chan et al., 2021), thus reducing the proteolysis of many intracellular proteins.

In addition, inhibitors of NFκB, AngII converting enzyme (ACE), or AT_1_R antagonists also indirectly inhibit MMP-2 activity in the vasculature (Wang et al., 2010; Marchesi et al., 2008). In fact, treating 2K-1C rats with the NFκB inhibitor, pyrrolidine dithiocarbamate, inhibited vascular MMP-2 expression and activity and improved endothelial dysfunction and hypertrophic remodeling (Cau et al., 2011). Several interesting review articles have discussed about the inhibitory effects of these inhibitors in the activity of MMPs in the cardiovascular system (Odenbach et al., 2011; Wu and Schmid-Schönbein, 2011).

In the next sessions, we will discuss how the antioxidant strategies may modulate the mechanistic relationship between oxidative stress-induced MMP activation, particularly MMP-2, in cardiovascular remodeling of hypertension and their potential translational implications for preventing hypertension-associated cardiovascular dysfunction. Although several reviews have independently discussed oxidative stress, antioxidants and MMPs in hypertension, this review tried to focus on the mechanistic relationship between oxidative stress-induced MMP-2 (and −9) activation as a driver for cardiovascular remodeling and dysfunction in hypertension.

## 4 Methodology

A thorough literature search in the PubMed database was used to perform this narrative review. The search aimed to identify experimental and clinical studies investigating the linkage between oxidative stress, MMPs, antioxidants, cardiovascular remodeling and hypertension. The search strategy employed combinations of the following keywords using operators as (AND/OR): “oxidative stress,” “reactive oxygen and nitrogen species,” “antioxidants,” “MMP,” “MMP-2,” “hypertension,” “vascular remodeling,” “cardiovascular dysfunction,” “nitric oxide,” “endothelial dysfunction” and “vascular smooth muscle cells.” Clinical trials, original research articles using animal models and cell culture experiments, and also relevant reviews, published in English, were considered suitable. Studies investigating the regulation or activity of MMPs, cardiovascular remodeling and dysfunction, the role of oxidative stress in hypertension, or the impact of antioxidants on these processes were included. Exclusion criteria comprised studies that did not specifically address alterations associated with oxidative stress or MMP-related pathways, as well as conference abstracts, editorials, letters to the editor, and articles unavailable in full text. Priority was given to studies published between 2000 and 2026. However, seminal studies published before 2000 were also included when necessary to provide historical context and support key mechanistic concepts discussed in this review.

## 5 The benefits of antioxidants in attenuating MMPs activity and hypertension-induced maladaptive vascular remodeling and dysfunction

**FIGURE 4:**
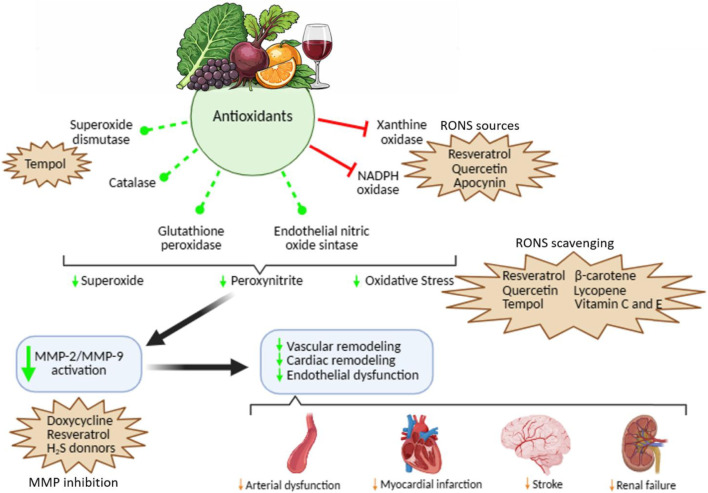
Mechanistic intervention points of antioxidant therapies in hypertension. Dietary and synthetic antioxidants may enhance the activity of key antioxidant enzymes, including superoxide dismutase, catalase, glutathione peroxidase and endothelial nitric oxide synthase (lines in green), while suppresses the activity of pro-oxidant enzymes such as NADPH oxidase and xanthine oxidase (lines in red), thereby decreasing RONs and MMP activation. Specific antioxidant compounds act at different stages of these pathways, ultimately attenuating cardiovascular remodeling, endothelial dysfunction and complications associated with hypertension, including myocardial infarction, stroke and renal failure.

Antioxidants can be categorized as enzymatic (such as CAT, GPx and SOD) and non-enzymatic scavengers, divided in either lipid-soluble (such as tocopherols, carotenoids and flavanols) or water-soluble (such as urate, ascorbate and glutathione) (Zehiroglu and Ozturk Sarikaya, 2019). In fact, growing number of evidence shows that many of the non-enzymatic antioxidants are present in the human vegetarian and Mediterranean diets. Vegetarian diet may reduce the risk of developing hypertension as it decreases the production of oxidant and inflammatory molecules and the circulating levels of MMP-9 when compared to an omnivorous-based diet (Navarro et al., 2016; Matsumoto et al., 2019; Szeto et al., 2004; C et al., 2016). Moreover, the Mediterranean diet, that is rich in white meat, fruits, vegetables and red wine (with lower intake of processed foods) may contribute to prevent the development of hypertension, diabetes and hyperlipidemia (Panche et al., 2016; Bakis et al., 2023). The number of antioxidants present in these diets reduces oxidative stress and enhances NO bioavailability, which may contribute to prevent vascular dysfunction (Goetz et al., 2016). We are describing below many antioxidants that also inhibit MMP activity in hypertension and others cardiovascular diseases. Figure 4 summarizes the effects of many antioxidants in ameliorating hypertension-induced other cardiovascular consequences.

### 5.1 Non-enzymatic, lipid-soluble scavengers

#### 5.1.1 Resveratrol

Resveratrol is one of the most important polyphenols of the Mediterranean diet and is notably found in grapes and red wine (Breuss et al., 2019). Resveratrol may exert its antioxidant effects by reacting and scavenging ONOO^−^, OH^−^ and O_2_^−^ in rat cardiomyocytes, murine macrophages (Leonard et al., 2003), rat coronary endothelial cells (Ungvari et al., 2007) and rat muscle cells (Hosoda et al., 2021). Resveratrol also decreases the activity of NADPH oxidase in diabetic rat endothelial cells via activation of sirtuin 1 (SIRT1)-induced deacetylation of NFκB (Bagul et al., 2015). Moreover, treatment of spontaneously hypertensive rats (SHR) and AngII-infused mice with resveratrol increased eNOS phosphorylation and NO production in the vasculature, which triggered flow-mediated vasodilation and prevented hypertension (Dolinsky et al., 2013). Likewise, treatment of SHR with resveratrol increased the expression of the catalytic β_1_ subunit of soluble guanylyl cyclase in the aorta, thus increasing cyclic guanosine monophosphate (cGMP) and vasodilation (Bhatt et al., 2011). Resveratrol also upregulated the GTP cyclohydrolase-1 in aortas of apolipoprotein E knockout mice, a rate-limiting enzyme of the biosynthesis of BH_4_, thus contributing to prevent eNOS uncoupling (Li and Forstermann, 2014; Xia et al., 2010). Resveratrol also upregulated SOD1 and 2 via Forkhead box O (FOXO)1 in cultured endothelial cells (Zhang et al., 2017; Spanier et al., 2009). Resveratrol may contribute to induce SIRT1 activation and decrease RONs, von Willebrand factor, intercellular adhesion molecule (ICAM)1 and caspase-3 in endothelial cells and umbilical arteries from women with pre-eclampsia (Bueno-Pereira et al., 2022). In addition, it may increase the antioxidant capacity of human umbilical vein endothelial cells (HUVECs) incubated with plasma of gestational hypertensive women (Viana-Mattioli et al., 2020).

By reducing oxidative stress, resveratrol may also decrease the expression and activity of MMPs, as previously discussed. In this context, resveratrol notably attenuated oxidative stress and MMP activity in the middle cerebral arteries of hypertensive-aged mice and reduced the incidence of cerebral microhemorrhages (Toth et al., 2015). Resveratrol reduced M/L and maladaptive hypertrophic remodeling of the resistance arteries in SHR (Behbahani et al., 2010). Resveratrol inhibited the phosphorylation of FAK and attenuated MMP-2 and -9 activities in VSMC exposed to high glucose, thus reducing migration (Lin et al., 2014). When resveratrol is given to SHR dams during gestation, it attenuated endothelial dysfunction and the development of hypertension in the adult offspring (Care et al., 2016). In the same context, resveratrol attenuated the over production of O_2_^−^ and improved cardiac function in adult male and female rat offspring submitted to intrauterine growth restriction caused by prenatal hypoxia and postnatal high-fat diet (Shah et al., 2017; Shah et al., 2016).

Several authors also discussed the beneficial effects of resveratrol in the heart, which include the activation of SIRT1 and reduction of the activity of NADPH oxidase. In fact, in the hearts of diabetic rats, resveratrol stimulated SIRT1-induced deacetylation of NFκB and reduction of the transcription of NADPH oxidase, which ameliorated hypertrophy (Bagul et al., 2015). When SIRT1 was overexpressed in mice cardiomyocytes, it upregulated FOXO-1 and SOD, thus resulting in increased antioxidant defense (Hsu et al., 2010) and reduction of cardiac hypertrophy and dysfunction (Alcendor et al., 2007). Resveratrol decreased MMP-2 expression and collagen deposition in the heart, improving diastolic dysfunction and fibrosis induced by transverse aortic constriction (TAC) in mice (Sung et al., 2015a; Sung et al., 2015b). Resveratrol also inhibited cardiac activity of the immunoproteasome catalytic subunit (β1i, β2i and β5i) in TAC in mice, which then blocked the proteolysis of phosphatase and tensin homolog (PTEN), thus reducing maladaptive cardiac remodeling and hypertrophy (Gal et al., 2021; Chen et al., 2019; Zou et al., 2019).

In humans, the use of resveratrol is associated to its action in the metabolism. In fact, the use of resveratrol at 250 mg/daily for 3 months in patients with coronary heart disease and arteriosclerosis (Berman et al., 2017), or in patients with type 2 diabetes (Bhatt et al., 2012; Zhang et al., 2015) may contribute to reduce total cholesterol and triglycerides. Human macrophages derived from peripheral blood monocytes were treated with resveratrol *in vitro* and the analysis of gene expression showed upregulation of the reverse cholesterol transporters, such as peroxisome proliferator-activated receptor (PPAR) C, liver X receptor alpha and 27-hydroxylase and ATP-binding cassette transporter A1, which prevented accumulation of LDL and triglycerides in the blood (Voloshyna et al., 2012). Resveratrol also increased SIRT1-PPARγ pathway and the expression of its downstream genes in cultured human macrophages: the fatty acid synthase and acetyl-CoA carboxylase, which suggests its potential role in the cholesterol metabolism in humans (Voloshyna et al., 2012).

Despite its potent antioxidant effects in preclinical studies, the clinical use of resveratrol may be limited by its poor oral bioavailability. Although nearly 70% of an ingested dose is absorbed in the intestine, extensive first-pass metabolism in both liver and intestine results in its rapid glucuronidation and sulfation (Walle, 2011). As a result, plasma concentrations of free resveratrol remain low even after the administration of relatively high doses. Clinical studies have evaluated doses ranging from 5 mg to 5 g per day; however, no consensus has been reached regarding the optimal dosage required to achieve cardiovascular benefits. Furthermore, while experimental models of hypertension consistently demonstrate beneficial effects of resveratrol on oxidative stress, endothelial dysfunction and cardiovascular remodeling, clinical outcomes have been variable. Although some clinical studies have reported improvements in endothelial function and inflammatory biomarkers, other trials have failed to show significant reductions in blood pressure or cardiovascular benefits (Berman et al., 2017) (Salehi et al., 2018). Differences in the patient characteristics, comorbidities, concomitant therapies and disease severity may contribute to these inconsistencies. Further large-scale randomized controlled trials are warranted to establish the efficacy and clinical applicability of resveratrol in hypertension-related cardiovascular disease.

#### 5.1.2 Quercetin

Quercetin is another flavonoid, known for its antioxidant and anti-inflammatory effects, and is richly found in broccolis, apples, peppers, parsley, green tea and onions (Alameri et al., 2012). Quercetin ameliorates vascular dysfunction in many cardiovascular diseases, including hypertension (Montenegro et al., 2010). Incubation of isolated 2K-1C rat aortas and mesenteric arteries with quercetin caused dilation, as it stimulated eNOS phosphorylation and NO production, an effect that was reduced in the presence of L-NAME or denuded endothelium (Touyz and Schiffrin, 2004; Montenegro et al., 2010). SHR treatment with quercetin reduced SBP and improved aortic endothelial dysfunction through reducing p47^phox^ levels and excessive RONs (Sánchez et al., 2006; Larson et al., 2012). Furthermore, quercetin restored plasma nitrite and nitrogen species levels in aortas from 2K to 1C rats, thus ameliorating endothelial dysfunction (Montenegro et al., 2010). Chronic treatment with quercetin to rats also reduced elastic stress of coronary arterial wall and increased the dilatory capacity through NO (Monori-Kiss et al., 2017). Finally, quercetin inhibited TGF-β1 expression and collagen deposition in balloon-injured abdominal aorta in rat, thus inhibiting VSMCs proliferation, migration and neointimal hyperplasia (Huang et al., 2009).

Quercetin reduces excessive production of RONs in the vasculature of hypertensive rats and contributes to attenuate MMPs activity. In fact, our group showed that the treatment of 2K-1C rats with quercetin reduced oxidative stress and MMP-2 activity in the aortas and coronary arteries, thus reducing hypertrophic remodeling (Pereira et al., 2018; da Rocha et al., 2023). Red wine polyphenols, including quercetin, also reduced ONOO^−^-dependent activation of MMP-2 and VEGF in the vasculature of AngII hypertensive rats (Walter et al., 2008). In addition, quercetin decreased aneurysm incidence in a model of aneurysm in mice by inhibiting MMP-2 and -9 activation and preventing oxidative stress, inflammation and arterial remodeling and dysfunction (Wang et al., 2014). Quercetin reduced MMP-2 activity and maladaptive characteristics of left ventricle in doxorubicin-treated rats (Ba et al., 2015).

In clinical trials, a supplementation of hypertensive patients (both genders at stage 1 of hypertension) with quercetin aglycone (365 mg, twice/day) contributed to reduce SBP, diastolic and mean arterial pressure by 5%. However, these effects of quercetin were not related to the reduction of circulating or urinary RONs levels (Edwards et al., 2007). In addition, the use of quercetin from onions skin (162 mg/day for 6-weeks, separated by 6-weeks washout) may lower SBP in overweight-to-obese patients with hypertension in the ambulatory (Brüll et al., 2015). In obese hypertensive patients, quercetin (150 mg/day, per 6-weeks, separated by 5-weeks washout) possibly contributed to decrease SBP in addition to decrease plasma levels of atherogenic oxidised LDL (Egert et al., 2009). Supplementation of hypertensive patients with a combination of extracts from grape seed and skin (330 mg), green tea (100 mg), resveratrol (60 mg) and a blend of quercetin, ginkgo biloba and bilberry (60 mg) were associated with reduced diastolic blood pressure and increased urinary nitrate and nitrite concentrations (Biesinger et al., 2016). When the metabolites from these polyphenols were incubated in the human aortic endothelial cells for 24 h, in media containing insulin, insulin-stimulated eNOS phosphorylation and higher production of nitrate and nitrite (Biesinger et al., 2016). On the other hand, quercetin typically exhibits poor water solubility, and its oral bioavailability varies according to the administered chemical form. Following absorption, it undergoes extensive intestinal and liver metabolism, resulting in conjugated metabolites that may have different biological activities (Dabeek and Marra, 2019). Clinical trials have generally used doses ranging 150 and 1,000 mg/day. Although experimental studies steadily demonstrate antihypertensive, antioxidant and vasoprotective effects of quercetin, clinical findings have been more modest. Meta-analyses suggest a slight reduction in blood pressure among hypertensive individuals; however, not all studies have reported consistent cardiovascular benefits (Serban et al., 2016). Variations in the formulation and bioavailability may contribute to these discrepancies. These may have stimulated the development of novel delivery formulations, including phytosome- and nanoparticles (Serban et al., 2016). Further well-designed randomized controlled trials are needed to clarify the long-term cardiovascular benefits of quercetin in hypertensive patients.

#### 5.1.3 Doxycycline

In the previous session, we briefly discussed the effects of doxycycline as an inhibitor of MMPs activity in inflammatory, oxidative conditions. Doxycycline differs mechanistically from the conventional antioxidants because its primary inhibits MMP activity rather than decreases oxidative stress. Nevertheless, antioxidant effects have also been described and may contribute to its cardiovascular protective actions. Among all tetracyclines, doxycycline and minocycline are the most potent MMP inhibitors in mammals (Golub et al., 1987). Doxycycline directly binds to the zinc ion in the catalytic domain of MMPs, thus inhibiting it (Golub et al., 1998), in addition to downregulate the mRNA levels *in vitro* (Jonat et al., 1996). In experimental models of hypertension, doxycycline decreased aortic MMP-2 and -9 activities as well as reduced arterial maladaptive remodeling (Castro et al., 2008; Rizzi et al., 2009; Castro et al., 2010; Castro et al., 2011; Guimaraes et al., 2011). Despite promising findings in experimental models, clinical evidence supporting the use of doxycycline as an MMP inhibitor in cardiovascular disease remains limited, and the optimal dose and long-term safety are still unknown (Clemens et al., 2018). Doxycycline also has extra pharmacological characteristics beyond inhibiting MMPs activity as some studies showed its antioxidant properties and the amelioration of vascular dysfunction in hypertension. In fact, doxycycline indirectly inhibited protein levels and activity of MMP-2 and -9 by reducing RONs in aortas of hypertensive rats (Hoeben et al., 1998). Treatment of SHR with doxycycline reduced oxidative stress and attenuated MMPs activity in aortas, which contributed to reduce hypertension (Antonio et al., 2014). Doxycycline improved endothelial dysfunction in the aortas of 2K-1C rats by increasing NO bioavailability and reducing oxidative stress (Castro et al., 2012b). Doxycycline also directly scavengers RONs in polymorphonuclear neutrophilic leukocytes *in vitro* (Hoeben et al., 1998). Doxycycline contributes to suppress doxorubicin-induced oxidative stress and apoptosis in the mouse cardiomyocytes, thus preventing systolic dysfunction and ventricular remodeling (Lai et al., 2010).

#### 5.1.4 Apocynin

Apocynin is an active compound, isolated from the medicinal herb *Picrorhiza kurroa*, that reduces SBP and ameliorates endothelial dysfunction in animal models of hypertension (Graton et al., 2019). Apocynin decreased the expression of p47^phox^ of NADPH oxidase and oxidative stress in aortic endothelial cells from SHR, thus increasing NO bioavailability. As a result, apocynin restored the dilatory effect of acetylcholine in mesenteric arteries and aorta of SHR (Graton et al., 2018; Perassa et al., 2016). Moreover, treatment of SHR with apocynin increased plasma antioxidant capacity in addition to reduce the pressor effect of AngII in mesenteric arteries (Perassa et al., 2016). Apocynin also increased the expression of eNOS, p-eNOS (activation site Ser1177), nNOS, iNOS, sGC-α, sGC-β and AT_2_R in aortas and mesenteric bed from SHR, thus increasing NO and restoring vascular endothelium response (Graton et al., 2019; Graton et al., 2018; Perassa et al., 2016). In 2K-1C rats, apocynin attenuated increased SBP and oxidative stress, thus preventing the loss of dilatory effects of acetylcholine in aortas (Castro et al., 2009). In addition, in mice infused with AngII, apocynin for 14 days prevented the increased SBP and inhibited endothelial dysfunction and collagen accumulation in the media layer of small arteries (Virdis et al., 2004). Treating isolated rat arterioles with norepinephrine and AngII for 4 h showed increased RONs and MMP-2 activity, which resulted in hypertrophic remodeling. Apocynin prevented oxidative stress and MMP-2 activation, which were accompanied by reducing inward arteriolar remodeling and endothelial dysfunction (Martinez-Lemus et al., 2011). On the other hand, treatment of 2K-1C rats with apocynin for 8 weeks did not attenuate MMP-2 activity in aortas (Castro et al., 2009). Despite the consistent amount of data regarding the benefit effects of apocynin in animal models of hypertension, clinical evidence remains extremely limited. Apocynin undergoes rapid metabolism and exhibits limited oral bioavailability (Chandasana et al., 2015; Wang et al., 2013), which may limit its therapeutic use in humans. Consequently, its clinical application remains largely exploratory. To date, few human studies have evaluated apocynin-containing formulations, and these have primarily focused on non-cardiovascular conditions, such as osteoarthritis (Virdis et al., 2004). Therefore, additional pharmacokinetic investigations and well-designed clinical trials are required before the promising findings observed in experimental models can be translated into clinical practice.

#### 5.1.5 β-carotene, lycopene and vitamin E

Dark green leafy vegetables, colored fruits and vegetables and some roots are rich sources of carotenoids (Gebregziabher et al., 2023). The β-carotene reduces oxidative stress and the risk of developing heart diseases (Fiedor and Burda, 2014). In fact, treating SHR with β-carotene for 10 weeks reduced SBP and plasma levels of malondialdehyde (Fiorelli et al., 2014). In humans, higher blood levels of β-carotenes were associated with lower risk of developing hypertension and atherosclerosis in both young and elderly patients (Karpp et al., 2013; Li et al., 2019). However, these positive effects cannot be attributed only to β-carotenes since clinical trials and meta-analyses have failed to demonstrate consistent benefits of β-carotene supplementation for the prevention of cardiovascular diseases. In fact, a high dietary intake of fruits and vegetables rich in carotenoids remains consistently associated with favorable health outcomes, since may reflect the combined effects of multiple bioactive compounds rather than the β-carotene alone (Ferreira-Santos et al., 2021).

Lycopene is another potent natural antioxidant belonging to the carotenoid family and is usually found in tomato extract. Lycopene contributes to scavenger RONs and minimizes some cardiovascular risks. Daily treatment of young and adult SHR with lycopene, for 4 weeks, reduced hypertension and aortic oxidative stress (Ferreira-Santos et al., 2021). Engelhard et al. showed that patients with hypertension who received tomato extract (250 mg of Lyc-O-Mato for 8 weeks, followed by placebo for 4 weeks) showed reduced levels of thiobarbituric acid-reactive substances (TBARs) in addition to reduce SBP and diastolic blood pressure (Engelhard et al., 2006). Furthermore, lycopene decreases mRNA and protein expression of MMP-9 in human hepatoma specificity protein-1 cells, thus reducing their invasion capacity. In fact, lycopene inhibited both expression of NFκB, specificity protein (Sp)1 and the insulin-like growth factor 1 receptor, which are necessary for the transcription of MMP-9 (Livny et al., 2002; Seifried et al., 2007). Lycopene has been shown in meta-analyses to support a slight decrease in SBP particularly in people who already had hypertension (Cheng et al., 2017). On the other hand, some randomized clinical trials failed to demonstrate significant improvements in lipid profile, inflammatory biomarkers, or markers of cardiovascular risk following lycopene supplementation (Th et al., 2012). Although the antihypertensive effects of lycopene may be promising, the overall clinical evidence is limited by small number of trials and heterogeneity in the supplementation protocols. Further large clinical studies are needed to establish cardiovascular benefits outcomes.

Vitamin E (or α-tocopherol) is usually found in avocados and chestnuts, and it is known to decrease oxidative stress and increases NO bioavailability in vascular and renal tissues (Sen et al., 2010). A clinical trial showed that mild hypertensive patients who received oral supplementation with vitamin E (200 IU/day for 12–27 weeks) showed reduction in SBP (Boshtam et al., 2002; Ziegler et al., 2020). Studies also demonstrated that vitamin E reduces lipid peroxidation and improves endothelial function, further supporting its vasoprotective effects (Van Dam et al., 2003; Nguyen et al., 2021; Ashor et al., 2015). Yet, the beneficial effects of vitamin E are not universal. Some randomized clinical trials reported no significant effect of vitamin E in the blood pressure or cardiovascular outcomes in mildly hypertensive individuals, thus suggesting that its efficacy may depend on baseline oxidative stress, nutritional status or dosage (Cook et al., 2007; Miller et al., 2005). Furthermore, a large meta-analysis including approximately 300,000 participants from 50 randomized trials found no evidence that vitamin E supplementation reduces the incidence of major cardiovascular events, including myocardial infarction, stroke, or cardiovascular mortality (Myung et al., 2013). These findings highlight the discrepancy between promising experimental results and the limited clinical benefits in human studies.

### 5.2 Non-enzymatic, lipid- and water-soluble scavengers

#### 5.2.1 Hydrogen sulfide donors

The hydrogen sulfide (H_2_S), properly named dihydrogen sulfide or sulfane (Filipovic et al., 2018), is an endogenous molecule with a well-established cardiovascular protective property, that has emerged as a promising therapeutic strategy for preventing hypertension (Elrod et al., 2007; Calvert et al., 2009; Testai et al., 2015; Citi et al., 2021; Testai et al., 2023). It is endogenously produced by the cystathionine γ-lyase (CSE), cystathionine β-synthase (CBS) and 3-mercaptopyruvate sulfurtransferase (3-MST), that are broadly expressed and coexist in the cardiovascular tissue. The endogenously produced H_2_S, as well as various H_2_S donors of natural occurrence or H_2_S synthetic donors, have been widely reported to possess vasodilatory and antioxidant properties, thus contributing to modulate systemic blood pressure (Citi et al., 2021; Yang et al., 2008; Zhao et al., 2001; Eberhardt et al., 2014; Liu et al., 2017; Martelli et al., 2023). The H_2_S interacts synergistically with NO signaling pathway to enhance vasodilatory responses. This interaction involves the inhibition of phosphodiesterase (PDE)-5, thus leading to elevated levels of cGMP, which further promotes vascular relaxation (Liu et al., 2022). Interestingly, hypertension has been reported in CSE knockout mice, accompanied by impaired endothelium-dependent relaxation and reduced H_2_S production in plasma and tissues, thereby highlighting the essential role of H_2_S as a key modulator of vascular function (Yang et al., 2008; Laggner et al., 2007). H_2_S was found to directly interact with the zinc active center of ACE, leading to its activity inhibition in endothelial cells (Laggner et al., 2007). In addition, treatment of SHR with Zofenopril, an ACE inhibitor containing a thiol group, reduced SBP and improved endothelium-dependent relaxation via H_2_S release. These vascular benefits were not observed with Enalapril, an ACE inhibitor lacking a thiol group (Bucci et al., 2014). Furthermore, NaHS has been shown to decrease SBP in an animal model of hypertension as 2K-1C rats and SHR (Liu et al., 2017; Xue et al., 2015; Pernomian et al., 2024).

Besides its vasodilatory actions, H_2_S exerts antioxidant effects by directly scavenging RONs and by enhancing endogenous antioxidant defenses, including SOD, GPx and CAT (Munteanu et al., 2023). The H_2_S donors attenuated cardiac and vascular remodeling in hypertension, thus reducing coronary vessel thickening, interstitial fibrosis and endothelial-to-mesenchymal transition (Shi et al., 2007; Pernomian et al., 2023; de Queiroz et al., 2025; Pernomian et al., 2025). NaHS treatment in rats with cardiac overload also improved angiogenesis by enhancing VEGF and CD31 positive cells, while suppressed MMP-9 expression, reducing intracardiac fibrosis and mitigated the transition from compensatory hypertrophy to heart failure (Givvimani et al., 2011). Similarly, GYY4137 reduced oxidative stress and fibrosis in rat myocardium following I/R injury (Meng et al., 2015; Meng et al., 2016), whereas NaHS limited infarct size and post-ischemic dysfunction through mechanisms involving NO signaling in mouse (Sun J. et al., 2016).

An important mechanism underlying the cardiovascular protection afforded by H_2_S involves the post-translational modification, S-sulfhydration (Livny et al., 2002). This modification enhances eNOS activity (Altaany et al., 2014) and contributes to regulate MMP activity. In fact, MMP-2 is upregulated in VSMC from CSE-knockout mice (Yang et al., 2012), whereas H_2_S inhibited MMP-2 transcription through S-sulfhydration of the transcription factor Sp1 (Zhu et al., 2022). The NaHS reduces the activity of MMP-1, -2, -7, -9, -14 and −23 by directly S-sulfhydrating their cysteine switch motifs *in vitro* (Zhu et al., 2022). Consistent with these findings, our group demonstrated that NaHS and 4-CPI reduced cardiac MMP-2 activity and attenuated cardiac hypertrophy in 2K-1C hypertensive rats (Pernomian et al., 2024).

Although promising results have been reported in animal models of cardiovascular disease, clinical evidence using H_2_S donors remain limited. Randomized controlled trials using taurine supplementation have demonstrated reductions in blood pressure, improvements in vascular function and increases in circulating H_2_S levels in prehypertensive individuals and patients with type 2 diabetes (Li et al., 2025). Taurine is the most abundant semi-essential sulfur-containing amino acid. It is synthesized *in vivo* from cysteine in the presence of cysteine dioxygenase, but is also obtained through the diet, particularly from sources such as eggs, meat and seafood. A randomized clinical study demonstrated that hypertensive group supplemented with taurine (1.6 g daily for 12 weeks) had a decrease in the SBP and diastolic blood pressure compared with baseline and placebo. Additionally, taurine improved endothelium-dependent and independent vasodilation, followed by an increase in plasma H_2_S (Sun Q. et al., 2016). Mechanistically, treated hypertensive rats with an enriched diet with 2% taurine for 12 weeks had an increase in 50% of vascular CSE and CBS levels. Similarly, *in vitro* exposure of human or mouse mesenteric arteries to taurine upregulated CSE and CBS, and reduced calcium mobilization through inhibition of transient receptor potential channel (Sun Q. et al., 2016). Nevertheless, the number of clinical studies remains small and further investigations are required to confirm the long-term cardiovascular benefits of this strategy.

### 5.3 Non-enzymatic, water-soluble scavengers

#### 5.3.1 Vitamin C

Vitamin C (or ascorbic acid) is an important antioxidant present in citrus fruits, such as kiwi, oranges and strawberries; it is a water-soluble antioxidant that is not stored in the body, thus having its excess eliminated through the urine. The use of vitamin C has been associated to lower SBP and reduce the incidence of atherosclerosis in humans, yet, its benefits may be contradictory. Although some evidence indicate that vitamin C reduces SBP and the risks of developing cardiovascular diseases (Appel et al., 1997; Roncaglioni, 2001; Block et al., 2001; Gale et al., 1995), other studies lack to show its beneficial effects in hypertensive patients (Zehiroglu and Ozturk Sarikaya, 2019). Differences in the basal status of the individuals (related to the vitamins and oxidative stress), their characteristics, supplementation dose and duration of treatment may contribute to the variability observed among clinical studies. On the other hand, experimental data are promising. By incubating human VSMC with LDL isolated from the blood of healthy volunteers with vitamin C, LDL was less oxidized by metals, homocysteine and myeloperoxidase-derived HOCl. Moreover, vitamin C attenuated VSMC apoptosis induced by oxidized LDL (Siow et al., 1999). Vitamin C lessens the inflammatory response associated with atherosclerosis by preventing the overexpression of ICAM1 and by reducing the adhesion of monocytes in the vasculature (Li and Schellhorn, 2007). Vitamin C also protects intracellular glutathione in the presence of oxidized LDL (Ishii et al., 2014). Vitamin C restored the cytokines-induced oxidized BH_4_ in HUVECs, thus contributing to increase eNOS activity and NO production (Heller et al., 2001).

#### 5.3.2 Nitrite

The nitrate-nitrite-NO axis is an important pathway to be considered for the benefit effects of NO in the vasculature. Nitrite is obtained from the diet after eating green vegetables and roots such as beats. The nitrate is absorbed in the intestine and about 20%–30% is transported by the bloodstream (Lundberg et al., 2008; Kapil et al., 2010). Part of the nitrate returns to the oral cavity and forms nitrite, especially by mouth bacterial flora. When nitrite is in the stomach, it forms NO in the presence of low pH. NO is also converted into nitrous acid and nitrous anhydride. Currently, nitrates have been studied as adjuvants to control blood pressure in animal models of hypertension (Ormesher et al., 2018). In fact, treatment with nitrate (1 mmol kg/day) or nitrite (15 and 45 mg/kg) to 2K-1C or L-NAME hypertensive rats reduced SBP (Montenegro et al., 2014; Ling et al., 2016). Chronic sodium nitrite has a marked antioxidant effect in 2K-1C hypertensive rats as reduced the activity of NADPH oxidase in the aortas. As a result, there was a decrease in RONs levels and an increase in NO bioavailability and cGMP, which improved vascular dysfunction in hypertension (Montenegro et al., 2011). Furthermore, nitrite markedly inhibited MMP-2 activity in the aorta and prevented hypertrophic remodeling in 2K-1C rats regardless of changing blood pressure (Rizzi et al., 2019). In a rat model of reduced utero placental perfusion, nitrite lowered SBP and ameliorated aorta hyperreactivity to phenylephrine in addition to decrease MMP-2 activity (Martins et al., 2023).

In clinical trials, pregnant hypertensive women who received a daily dose of beetroot juice (400 mg nitrate) for 8 days during their second trimester showed significant increase in plasma and salivary nitrate/nitrite concentrations, which were negatively correlated with diastolic blood pressure (Ormesher et al., 2018). Pregnant hypertensive women appear to have a lower abundance of nitrate-reducing bacteria in the oral microbiota, which may impair the conversion of nitrate to nitrite and limit NO bioavailability. However, nitrate/nitrite levels increased in their saliva and plasma following acute supplementation with beetroot juice (Willmott et al., 2023). These findings support the relevance of the nitrate–nitrite–NO in the antihypertensive response to dietary nitrate supplementation in humans. However, larger randomized controlled trials are required to determine the long-term efficacy of nitrate-rich interventions in hypertensive patients.

#### 5.3.3 Tempol

Tempol is a synthetic, water-soluble antioxidant that mimics the activity of SOD. In a model of salt-sensitive hypertensive mice, treatment with tempol for 7 days reduced salt-induced increased SBP, RONs and the protein levels of the epithelial sodium channel in the kidney cortex (Chacko et al., 2021). Treatment of SHR dams during pregnancy and lactation with tempol reduced oxidative stress in the urine and lowered hypertension in the neonatal offspring (Kawakami et al., 2024). Moreover, tempol restored the impaired adenine nucleotide transfer and voltage-gated anion channel of the mitochondria and cardiac dysfunction in rats treated with TNF-α through reducing oxidative stress (Mariappan et al., 2007).

In 2K-1C rats, tempol reduced aortic oxidative stress and MMP-2 activity, which contributed to improve hypertrophic arterial remodeling and dysfunction (Castro et al., 2009). Tempol also reduced oxidative stress and the activation of MMP-2 by S-glutathiolation in the aortas of 2K-1C rats, thus decreasing the proteolytic effect of MMP-2 on calponin-1 (Blascke de Mello et al., 2019). In addition, tempol reduced RONs levels and MMP-2 activity in rat cremaster arterioles, previously pressurized *in vitro* and incubated with noradrenaline and AngII. Tempol significantly reduced the inward arterial remodeling caused by noradrenaline and AngII *in vitro* (Martinez-Lemus et al., 2011). On the other hand, TGF-β is activated by oxidative stress in hypertension and regulates gene expression of type I collagen and MMP-2 in the heart, thus contributing to fibrosis (Mariappan et al., 2007). Tempol reduced the levels of TGF-β, oxidative stress and MMP-2 activity in the hearts of 2K-1C rats, thus improving cardiac contractile dysfunction and hypertrophy (Rizzi et al., 2013). Finally, by testing tempol in the reactivity of isolated small arteries from normotensive and hypertensive individual, it increased vasodilation in response to acetylcholine, thus improving endothelial function (Bruno et al., 2017). Although there is a vast literature in cell culture systems and animal models concerning the benefit effects of tempol in reducing oxidative stress and endothelial dysfunction, clinical trials are currently lacking and warrants further investigation.

Figure 4; Table 1 summarize the impact of using antioxidants as a pharmacological mechanism of intervention to reduce oxidative stress, MMP-2 activity and cardiovascular dysfunction in hypertension and its cardiovascular alterations. Furthermore, Table 1 illustrates the clinical evidences of these antioxidants and their limitations.

**TABLE 1:** An overview of the antioxidant intervention that include their main molecular targets, type of matrix metalloproteinases (MMPs) they affect, experimental models, clinical evidence, and the general degree of evidence regarding cardiovascular remodeling and dysfunction associated with hypertension.

Intervention	Molecular targets	MMPs	Type of MMP modulation	Experimental models	Clinical evidence	Main limitations
Resveratrol	SIRT1; NFκB; NADPH oxidase; eNOS	Reduces MMP-2 and -9 expression and/or activity and attenuates cardiovascular remodeling and dysfunction in experimental models	Indirect	SHR; AngII-infused mice/rats; DOCA-salt hypertension; 2K-1C	Moderate	Low oral availability; divergent clinical results; divergence of dose
Quercetin	NADPH oxidase; eNOS; NO signaling; TGF-β-and anti-inflammatory pathways	Reduces MMP-2 activity and attenuates vascular and cardiac remodeling	Indirect	SHR; 2K-1C rats; Ang-II-induced hypertension	Moderate	Low availability; divergent clinical results
Apocynin	NADPH oxidase inhibition; eNOS; NO-cGMP pathway	Prevents oxidative stress-induced MMP-2 activation and vascular remodeling	Indirect	SHR; 2K-1C rats; AngII-infused mice; isolated arterioles	Very limited	Scarcity of human studies
β-Carotene	RONs scavenging; lipid peroxidation attenuation	No evidence regarding MMP regulation identified in hypertension models	Not established/Unknown	SHR	Weak	No convincing evidence for cardiovascular protection in supplementation trials
Lycopene	NF-κb; LDL oxidation; redox transcriptional pathways	Decreases MMP-9 expression through inhibition of transcriptional pathways	Indirect	SHR	Moderate	Small effect size; heterogenous clinical outcomes
Vitamin E	Lipid peroxidation pathways; antioxidant system; NO bioavailability	No direct evidence of MMP modulation reported	Not established/Unknown	SHR; DOCA-salt rats	Conflicting	Meta-analyses failed to show cardiovascular protection
Vitamin C	BH4 preservation; favors eNOS coupling; decreases oxidized LDL; antioxidant mechanisms	No direct evidence of MMP modulation reported	Not established/Unknown	Human vascular smooth muscle cells (VSMCs); human umbilical vein endothelial cells (HUVECs)	Conflicting	Contradictory evidence regarding blood pressure reduction and cardiovascular benefits
H_2_S Donors	CSE/CBS/3-MST pathway; eNOS; Sp1 sulfhydration; NO signaling	Inhibits several MMPs (−1, −2, −7, −9, −14, −23) activities by sulfhydration of cysteine in their catalytic site and suppresses MMP-2 transcription through Sp1 modification; reduces cardiovascular remodeling and fibrosis	Direct and Indirect	SHR; 2K-1C rats; CSE knockout mice; *ex vivo* myocardial ischemia/reperfusion injury and transverse aortic constriction pressure-overload models	Limited	Few human studies; lack of long-term cardiovascular outcomes
Dietary Nitrite	Nitrate–nitrite–NO pathway; NADPH oxidase; cGMP signaling	Reduces MMP-2 activity and prevents vascular hypertrophic remodeling	Indirect	2K-1C rats; L-NAME hypertension; reduced uterine perfusion pressure model	Moderate	Dependence on oral microbiota and pH
Tempol	Superoxide scavenging; SOD mimetic; TGF-β signaling	Reduces MMP-2 activity and oxidative activation mechanisms associated with vascular and cardiac remodeling	Indirect	2K-1C rats; SHR; salt-sensitive models; isolated arterioles	Absent	Absence of clinical trials
Doxycycline	Inhibition of catalytic zinc-binding site of MMPs; indirect antioxidant effects	Directly inhibits the catalytic domain of MMPs, thus inhibiting their activity	Direct	SHR; 2K-1C rats; DOCA-salt hypertension	Limited	Optimal dosing unknown; few cardiovascular trials

Abbreviations: Ang II, angiotensin II; BH4, tetrahydrobiopterin; CBS, cystathionine beta-synthase; c-GMP, cyclic guanosine monophosphate; CSE, Cystathione y-lyase; DOCA, deoxycorticosterone acetate; eNOS, endothelial nitric oxide synthase; H_2_S, hydrogen sulfide; LDL, low-density lipoprotein; L-NAME, N-nitro-L-arginine methyl ester; MMP, matrix metalloproteinase; NADPH, oxidase, nicotinamide adenine dinucleotide phosphate oxidase; NF-kB, nuclear factor kappa B; NO, nitric oxide; RONs, reactive oxygen and nitrogen species; SIRT1, sirtuin 1; SHR, spontaneously hypertensive rat; SOD, superoxide dismutase; Sp1, specificity protein 1; TGF- β, transforming growth factor beta; 2K-1C, two-kidney, one-clip renovascular hypertension model; 3-MST, 3-mercaptopyruvate sulfurtransferase.

## 6 Conclusion and perspectives

The use of antioxidants as adjunctive therapies for hypertension and related cardiovascular diseases may gain an increasing attention due to the central role of oxidative stress in promoting MMP activation, cardiovascular remodeling and dysfunction. The evidence summarized in this review supports the concept that reducing oxidative stress may attenuate MMP-mediated vascular and cardiac alterations, thereby helping to complement current antihypertensive therapies. However, despite robust experimental findings, clinical translation of antioxidant-based interventions remains challenging because of heterogeneous outcomes across studies and the complexity of redox regulation in humans, and the frequent discrepancy between preclinical efficacy and clinical effectiveness.

Future research should move beyond the generalized use of antioxidants and focus on identifying specific redox-sensitive mechanisms that regulate MMP activity in hypertension. In particular, further investigation is warranted into post-translational modifications involved in MMP-2 activation, including S-glutathiolation and S-sulfhydration, as potential therapeutic targets. In addition, the development of biomarkers capable of monitoring oxidative stress and MMP activity may help identify patient populations most likely to benefit from antioxidant interventions. Well-designed clinical trials should also evaluate cardiovascular remodeling, vascular function and MMP-related endpoints, rather than blood pressure alone, to better define therapeutic efficacy.

Another important priority is the development of targeted antioxidant strategies capable of restoring pathological redox imbalance without disrupting physiological redox signaling. Such approaches may include compounds that selectively modulate NADPH oxidase-derived oxidative stress, enhance endogenous antioxidant defenses or directly interfere with MMPs activation pathways. A deeper understanding of the interplay among oxidative stress, MMPs and cardiovascular remodeling will facilitate the development of precision therapies and may support the integration of redox-based interventions into future cardiovascular disease management.
